# Deciphering the role of transcription factors in glioblastoma cancer stem cells: Transcription factors in glioblastoma stem cells

**DOI:** 10.3724/abbs.2024061

**Published:** 2024-05-08

**Authors:** Kaishu Li, Haichao Li, Aonan He, Gengqiang Zhang, Yuyao Jin, Junbin Cai, Chenle Ye, Ling Qi, Yawei Liu

**Affiliations:** 1 Department of Neurosurgery & Medical Research Center Shunde Hospital Southern Medical University (The First People’s Hospital of Shunde Foshan) Foshan 528300 China; 2 Department of Neurosurgery Nanfang Hospital Southern Medical University Guangzhou 510515 China; 3 Institute of Digestive Disease Affiliated Qingyuan Hospital Guangzhou Medical University Qingyuan People’s Hospital Qingyuan 511518 China; 4 Department of Neurosurgery Affiliated Qingyuan Hospital Guangzhou Medical University Qingyuan People’s Hospital Qingyuan 511518 China

**Keywords:** transcription factors, cancer stem cells, glioblastoma, therapeutic strategy

## Abstract

Glioblastoma (GBM), the most aggressive and fatal brain malignancy, is largely driven by a subset of tumor cells known as cancer stem cells (CSCs). CSCs possess stem cell-like properties, including self-renewal, proliferation, and differentiation, making them pivotal for tumor initiation, invasion, metastasis, and overall tumor progression. The regulation of CSCs is primarily controlled by transcription factors (TFs) which regulate the expressions of genes involved in maintaining stemness and directing differentiation. This review aims to provide a comprehensive overview of the role of TFs in regulating CSCs in GBM. The discussion encompasses the definitions of CSCs and TFs, the significance of glioma stem cells (GSCs) in GBM, and how TFs regulate GSC self-renewal, proliferation, differentiation, and transformation. The potential for developing TF-targeted GSC therapies is also explored, along with future research directions. By understanding the regulation of GSCs by TFs, we may uncover novel diagnostic and therapeutic strategies against this devastating disease of GBM.

## Introduction

Glioblastoma (GBM) is one of the most common and highly invasive types of heterogeneous glioma tumors encountered clinically. The 5-year survival rate is very low, reflecting a high recurrence rate of more than 90% for GBM, and despite adjuvant therapy with temozolomide (TMZ) chemotherapy and radiotherapy (RT), the prognosis remains poor [1]. Tumor recurrence is closely related to stem cell-like characteristics in the tumor region [2]. Cancer stem cells (CSCs), an abnormal and uncontrolled subgroup of tumor cells known for their self-renewal and multilineage differentiation abilities, contribute to the formation of various tumor cell types [3]. Gliomas have two cell types: glioma stem cells (GSCs) and differentiated tumor cells [4]. Notably, some commonalities can be observed in GSCs within GBM, such as self-renewal, nondifferentiation, tumor invasion, and drug resistance, as well as differences in marker expression and differentiation potential [5]. These intrinsic limitations of GSCs have become obstacles in the field of GBM treatment. 

In the human genome, there are at least 1600 transcription factors (TFs), approximately 19% of which are related to disease phenotypes [6]. TFs regulate nearly the entire genome through one domain that binds to a specific DNA sequence and another that binds to protein coactivators or corepressors [ 6, 7] . The phenotypic characteristics of GBM are mediated by a series of signaling pathways and mutations, and TFs are instrumental in controlling genes that govern GSC maintenance, differentiation, and tumorigenicity. TFs adjust gene expression in response to various intracellular and extracellular environments (hypoxia) and signals [epithelial–mesenchymal transition (EMT), cell cycle, apoptosis, metabolic reprogramming]. Importantly, they facilitate the interactions of CSCs with their surrounding microenvironment, including their stemness, matrix, and immune system [8]. Furthermore, highly specific TFs usually only regulate a limited set of gene targets; hence, inhibitors of such TFs are less likely to affect compensatory drug resistance mechanisms common to many drugs. Currently, strategies for effectively treating diseases by targeting abnormal TFs, which involve disrupting multiple attributes of tumor cells through the blockade of TFs, ultimately leading to tumor regression, have been proposed. Unsurprisingly, considerable effort and resources have been invested in identifying small molecules that can effectively and specifically inhibit TFs. Therefore, inhibitors related to TFs in GBM have a wide range of clinical applications ( Table 1).

**Table:** **Table 1** Preclinical/clinical trials targeting TF inhibitors of GSCs

TF	Inhibitor	Combination	Mechanism of action		Clinical trial/Ref.	Year	Status
Sox2	Rapamycin	TMZ, Dox	Rapamycin decreases SOX2 expression and TMZ resistance.		NCT03463265	2018	Completed, has results
Cyclopamine	NA	Cyclopamine decreases SOX2 expression.		[9]	2016	Preclinical research
Oct4	VP	PDT	VP affects YAP-TEAD signaling pathway in human glioma cells, down-regulates VEGFA expression and pluripotency marker Oct-4 in human glioma cells to inhibit the growth of glioblastoma cells.		NCT04590664	2021	Recruiting
Nanog	NANEP	NA	NANEP5 is a chimeric dominant repressor that acts by blocking endogenous NANOG function.		[10]	2019	Preclinical research
c-MYC	Omomyc	NA	Omomyc targets c-MYC function to reduce the proliferation of glioblastoma cells.		[11]	2021	Preclinical research
HIF-1	LBH589	Scriptaid+ Delta24-RGD, DZ‑NEP+TMZ	LBH589 inhibits glioblastoma growth and angiogenesis through suppression of HIF-1α expression.		NCT00859222	2009	Completed, has results
NF-κB	BAY 11-7082	NA	NF-κB inhibitor BAY 11-7082 suppresses the expression of MGMT and enhances the TMZ-induced apoptosis in TMZ resistant U251 cells to treat TMZ resistant glioblastoma.		[12]	2020	Preclinical research
Sulfasalazine	NA	Sulfasalazine triggers the apoptosis in glioma cells by inhibiting action of NF-κB.		NCT04205357	2020	Completed
STAT3	ODZ10117	NA	ODZ10117 inhibits STAT3 activation in glioblastoma cells, targeting the SH2 domain of STAT3, resulting in inhibition of STAT3 tyrosine phosphorylation, dimerization, nuclear translocation, and transcriptional activity, thereby reducing stem cell properties.		[ 13, 14]	2019	Preclinical research
β-catenin	SEN461	NA	SEN461 protected AXIN degradation, causing β-catenin loss to suppress WNT signaling in GBM cells which could inhibit the growth of GBM cells.		[ 15, 16]	2013	Preclinical
YAP/TAZ	Verteporfin	PDT	Verteporfin binds to the conserved TEAD interaction domain in YAP, disrupts YAP-TEAD binding, and induces YAP/TAZ protein degradation, preventing transcriptional transactivation.		NCT04590664	2021	Recruiting

VP, verteporfin; PDT, photodynamic therapy; Dox, Doxorubicin; NA, not applicable; DZ‑NEP, 3-deazaneplanocin A; TMZ, temozolomide; Data sources- ClinicalTrials.gov ( https://clinicaltrials.gov).

This review comprehensively describes the origin of TFs, the master regulators of GSCs in complex tumor microenvironments, and the current knowledge of abnormal TF activities in GBM, including the potential interaction mechanisms that may exist between dysregulated TFs and GBM. This review also summarizes the current status of therapeutic measures against GBM tumors targeting TF inhibitors, providing practical clinical reference value for the current development of new drug targets for GBM tumors.

## Stem Cells Drive the Onset of GBM

**Figure 1:**
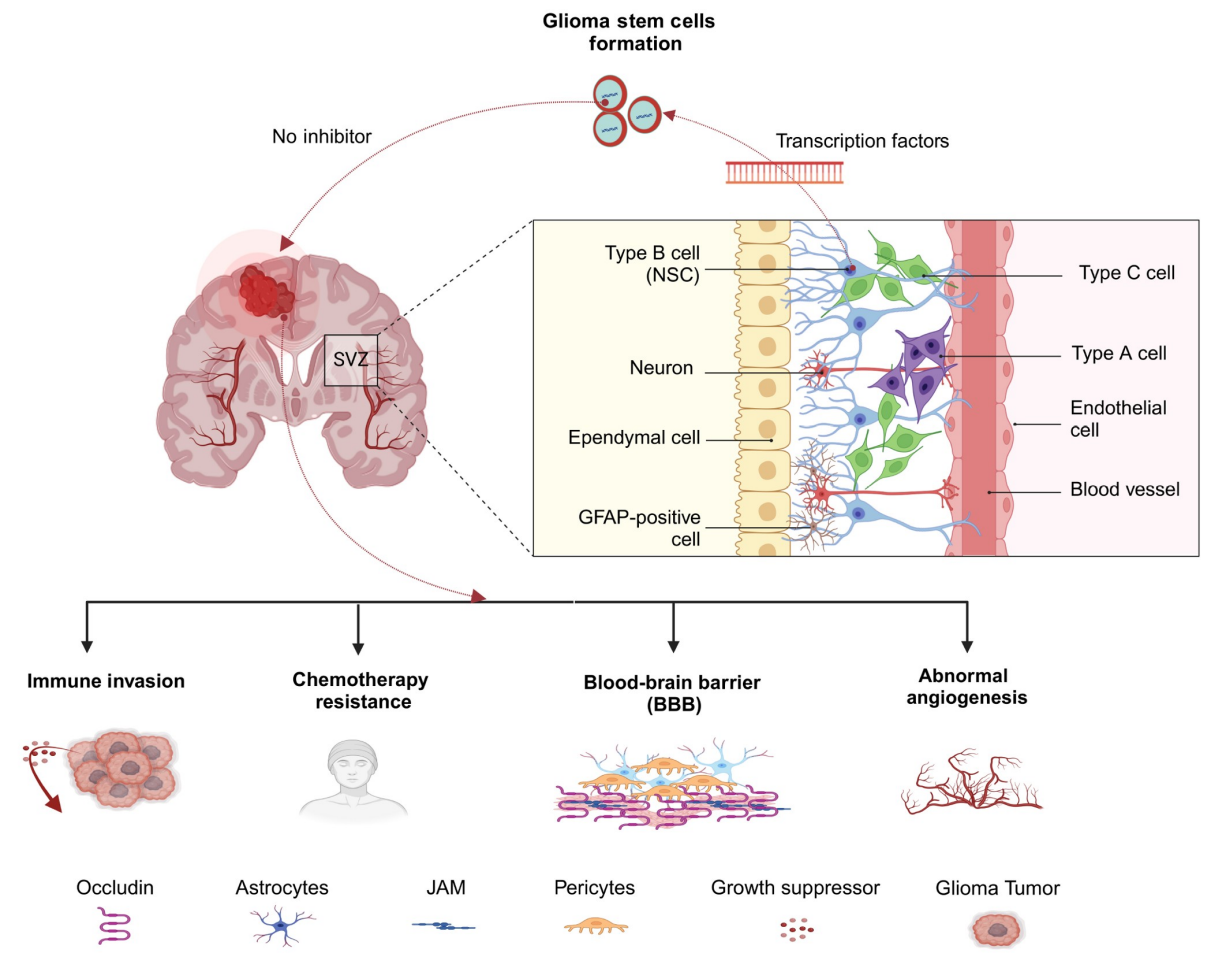
Figure 1 Transcription factors play a crucial role in the origin and development of glioma stem cell Type A cell: migrating neuroblast; Type B cell: neural stem cell (NSC); Type C cell: transit amplifying progenitor cell; GFAP: glial fibrillary acidic protein.

The subventricular zone (SVZ) is the largest neurogenic niche in the adult central nervous system and is closely associated with the pathogenesis of GBM [17]. A substantial body of research supports the notion that neural stem cells (NSCs) within the SVZ may serve as potential cells of origin for GBM. There are many similarities between NSCs and GBM, including low-level mutations in telomerase reverse transcriptase (TERT) and certain oncogenes, such as epidermal growth factor receptor (EGFR), phosphatase and tensin homolog (PTEN), and tumor protein p53 (TP53) [ 17, 18] . Both cell types also exhibit stem cell-like characteristics, such as self-renewal, differentiation, and the ability to form neurospheres, and they coexpress stemness markers such as CD133, Sox2, and Nestin [ 19– 22] . Conversely, GSCs differ from NSCs in their spectrum of gene mutations, chromosomal abnormalities, and tumorigenicity [23]. TFs are expressed at relatively high levels in GSCs, maintaining the perpetual self-renewal of GBM cells [24]. Typically, NSCs in the SVZ do not express IDH1, but certain NSCs carrying low-frequency mutations in IDH1 and TP53 can migrate outward, eventually progressing to tumor sites distant from the SVZ. During this migration, high levels of TFs (such as TP53, FOXG1, SOX2, and c-MYC) accumulate, mediating the transformation of NSCs into GSCs with oncogenic potential [ 17, 24– 26] . Research by Andromidas *et al*. [27] further revealed that the NSCs in the SVZ that transform into the GBM are primarily GFAP ^+^ cells. Moreover, studies indicate that the GBM recurrence rate is greater and the prognosis is poorer in regions surrounding the SVZ, which may be related to the NSCs within the SVZ [ 28– 30] . This may be because the SVZ has a richer vascular supply, and the NSCs within the SVZ can secrete chemotactic factors such as chemokine ligand 12 (CXCL12), which induces GBM to migrate toward the SVZ area, while the microenvironment of the SVZ may promote the dedifferentiation of GSCs [ 31, 32] . However, there are also studies reporting that neuroglial cells and astrocytes may serve as the origin of GSCs, with astrocytes and GBM both expressing GFAP and being able to interact through the NF-κB signaling pathway [ 33, 34] . Although the origin of GSCs remains controversial, the view that stem cells within the SVZ are an important source of GSCs has been widely accepted. Regardless of their origin, GSCs remain the driving force behind GBM recurrence and resistance to therapy. A schematic diagram of the hypothesis of GSC origin and development is shown in Figure 1.

## TFs Regulate and Influence GSCs

TFs are instrumental in the biological regulation of CSCs/GSCs, orchestrating the expressions of genes associated with stemness, self-renewal, differentiation, and tumorigenicity [ 35, 36] . Studies have identified TFs associated with migration and invasion in cultured GSCs or patient-derived xenograft glioma models. However, disambiguation of these TFs has been difficult to achieve to a large extent because invasive tumor cells adapt in complex ways to motility, adhesiveness, hypoxia, metabolism, and immune responses within their microenvironments [37]. Currently, in addition to the classical TFs involved in GBM, including SOX2, OCT4, NANOG, KLF4, c-MYC, β-catenin, STAT3, NF-κB, HIF-1α and YAP/TAZ [ 38– 40] , there are also numerous other GSC-related TFs ( Table 2). Understanding the complex interplay between GSCs, their unique characteristics, and the regulatory role of TFs is challenging yet crucial.

**Table:** **Table 2** Other research in GSC-related TFs

Marker	Function		Experimental evidence	Type	Ref.
CD133	Facilitating self-renewal and tumorigenicity, serves as a significant prognostic indicator for overall survival and progression-free survival in patients with GBM.		FACS, xenotransplantation	Cell surface marker	[ 41, 42]
CD44	It is involved in cell adhesion and is associated with invasion, proliferation, migration and self-renewal of GBM stem cells.		FACS, xenotransplantation	Cell surface marker	[ 43, 44]
CD15	It is associated with the stemness and differentiation of GBM stem cells.		FACS, xenotransplantation	Cell surface marker	[ 45, 46]
S100A4	Participating in the self-renewal of GBM stem cells and associated with proliferation, an upstream regulator of the mesenchymal program.		FACS, xenotransplantation	Intracellular marker (Protein)	[47]
A2B5	Closely associated with the stemness of GBM stem cells, promoting the proliferation and migration/invasion of GBM.		FACS, xenotransplantation	Cell surface marker	[ 48, 49]
Nestin	It is associated with the cell cycle and invasion/migration of GBM stem cells.		FACS, xenotransplantation	Intracellular marker (Protein)	[ 50, 51]
Olig2	Critical for the formation and proliferation of gliomas.		FACS, xenotransplantation	Intracellular marker (TF)	[52]
BMI1	Blocking the immunogenicity and differentiation of GBM stem cells, as well as participating in the migration and invasion of CD133 ^+^ cells.		FACS, xenotransplantation	Intracellular marker (TF)	[ 53, 54]
EFGR	Promoting angiogenesis and invasion and associated with self-renewal and proliferation.		FACS, xenotransplantation	Cell surface marker	[ 55, 56]
TP53 (p53)	Critical for the survival of GBM stem cells, and promotes the proliferation, migration/invasion, and self-renewal capacity of GBM stem cells.		FACS, xenotransplantation	Intracellular marker (TF)	[ 57, 58]
PTEN	Tumor suppressor gene, its loss leads to self-renewal of tumor proliferation.		FACS, xenotransplantation	Intracellular marker (Protein?)	[ 59, 60]
Musashi-1	Regulating the cell cycle and DNA replication, simultaneously crucial for cell proliferation and migration, while promoting the expression of CD44.		FACS, xenotransplantation	Intracellular marker (Protein)	[ 61– 64]
L1CAM	Regulating the DNA damage response of GBM stem cells through NBS1 and associated with the survival and radiotherapy resistance of GBM.		FACS	Cell surface marker	[ 65, 66]
IDH1/IDH2	A typical GBM biomarker, it is a rate-limiting enzyme in the Krebs cycle, promoting the formation of the tumor microenvironment.		NA	Intracellular marker (Enzyme)	[67]
Integrin α6	Capable of enriching the population of GBM stem cells, facilitating the self-renewal and proliferative capacity of GBM stem cells.		xenotransplantation	Cell surface marker	[68]
SSEA-1	SSEA-1 ^+^ cells exhibit higher proliferative capacity and are closely associated with tumorigenicity.		FACS, xenotransplantation	Cell surface markers	[69]

NA, not applicable; FACS, fluorescence cell sorting.

### SOX2

SOX2 is a transcription factor that maintains the regenerative capacity and pluripotency of undifferentiated NSCs and specific tumor cell subgroups, including GBM stem cells. The expression of SOX2 is typically highly restricted in the adult brain but is induced to high levels in GBM [ 70, 71] . The accumulation of high levels of SOX2 in NSCs in the SVZ may be associated with the occurrence of GBM, but currently, there are no studies on its specific mechanisms [24]. The relationship between SOX2 and the development of GBM is quite complex. Lopez-Bertoni *et al* . [71] linked SOX2 to GSCs and reported that SOX2 induces GSC stemness and proliferative capacity by inhibiting ten-eleven translocation 2 (TET2) and regulating the modification of 5-hydroxymethylcytosine (5hmC) in DNA. K-M survival analysis of 40 clinical GBM patients revealed that abnormal expression of SOX2 is associated with a poorer prognosis in GBM patients [72]. The poor prognosis of GBM patients is likely related to the drug resistance caused by SOX2. It has been reported that SOX2 may induce chemotherapy resistance by promoting EMT, ATP-binding cassette drug transporters, antiapoptotic and/or prosurvival signaling, lineage plasticity, and evasion of immune surveillance [ 73, 74] . However, Garros-Regulez *et al*. [9] improved the resistance of GBM to TMZ by inhibiting mTOR with rapamycin and reducing the expression of SOX2, reversing the poor prognosis of GBM. Oppel *et al*. [75] reported that SOX2 can regulate the migration and invasion of GBM cells through the RhoA-dependent pathway and focal adhesion kinase (FAK) signaling. Furthermore, SOX2 is involved in four major signaling pathways—TGF-β, SHH, EGFR, and FGFR, and is regulated by these pathways, affecting the progression of GBM [76]. SOX2 is central to the regulation of GBM stemness and malignancy. 

### OCT4

OCT4, also known as POU5F1, is a key transcription factor that maintains the pluripotency and self-renewal of ESCs [77]. Although its expression is typically absent in normal somatic cells, it is present in germline fibroblasts, ESCs, and a subset of cancer cells [78]. Inhibition of OCT4A expression can suppress the proliferation and self-renewal of GSCs [79]. Furthermore, OCT4 can also induce the transformation of GBM cells into GSCs by activating the *DNMT* promoter, leading to DNA methylation. Within GSCs, OCT4 can regulate cytokine/chemokine signaling and cause immune evasion by inhibiting the infiltration and function of T cells [ 80, 81] . Ikushima *et al*. [82] and Smith *et al*. [ 83] reported that knockdown of *OCT4* can effectively enhance the sensitivity of GSCs to TMZ. High levels of OCT4 expression have been associated with poor prognosis and lower survival rates in patients with GBM and glioma, likely due to the role of OCT4 in activating the NF-κB/PI3K/AKT pathways and the downstream interregulation of target gene products such as SOX2 and BMI1 [ 84– 86] . In summary, OCT4 plays a pivotal role in regulating the biological functions of GBM and glioma GSCs, influencing their stemness, tumorigenicity, therapeutic resistance, and metastatic potential. 

### NANOG

The expression of NANOG, a stem cell transcription factor, gradually decreases during the differentiation of embryonic stem cells, and NANOG is generally not expressed in somatic cells; however, its expression is upregulated in CSCs [ 87, 88] . To verify the role of NANOG in GSCs, shRNA-mediated knockdown of *NANOG* revealed that it can affect the stemness of GSCs through the HH-GLI signaling pathway, regulate the proliferation of GBM via the PI3K/AKT pathway, and cause GSCs to arrest in the G0/G1 phase of the cell cycle [ 89– 91] . The role of NANOG in GBM is further complicated by its interaction with other genes and pathways, such as the IL6/JAK2/STAT3 pathway, NANOG-CXCR4 pathway and NANOG/SOX2/CD133 axis, which are crucial for maintaining the stemness and tumorigenic properties of GSCs [ 90, 91] . The expression level of NANOG is not only positively correlated with pathological grade but can also serve as a prognostic factor for survival [ 92, 93] . Although inhibiting NANOG expression does not affect the expression of MGMT, it can reduce the drug resistance of GBM, and the poor prognosis of GBM is likely related to the drug resistance caused by NANOG [91]. Intriguingly, NANOG has also been associated with resistance to hormonal therapy and chemotherapy [94]. In summary, these findings support Nanog as a potential therapeutic target for the treatment of GBM tumors. 

### c-MYC

The c-MYC oncoprotein is a DNA-binding TF that requires heterodimerization with MYC-associated factor X (MAX) to activate transcription and binding to E-box sequences. The unregulated activity of c-MYC in cancer cells induces the transcription of genes involved in adaptation to hypoxia, such as genes involved in angiogenesis [95]. MYC directly controls the expressions of glucose metabolism genes, such as glucose transporter (GLUT1) and hexokinase 2 (HK2), stimulating the Warburg effect [96]. Amplification of EGFR can lead to heterogeneous nuclear ribonucleoprotein A1 (hnRNPA1)-dependent selective splicing of the MYC-interacting partner MAX, producing the functionally enhanced protein δMAX, which promotes GBM glycolytic metabolism in a MYC-dependent manner [96]. Inhibition of MYC triggers mitotic catastrophe in GBM cells, causing cell cycle arrest at the G0/G1 phase and increased apoptosis [ 64, 65] . Additionally, c-MYC can maintain the stemness and tumorigenicity of GSCs through cell cycle regulation [97]. Clearly, c-MYC plays a complex role in the tumor microenvironment of GBM. c-MYC is dysregulated in 70% of tumors, and targeting the dysregulated MYC protein plays a broad therapeutic role. However, c-MYC is located primarily in the cell nucleus, and targeting nuclear c-MYC with specific monoclonal antibodies is technically very challenging. Moreover, due to the lack of small molecule-specific active sites, it is difficult to inhibit its activity using strategies similar to those used for kinases [98]. Therefore, the design of GBM treatment strategies targeting c-MYC requires careful consideration. 

### NF-κB

NF-κB is a protein complex that governs the transcription of DNA, cytokine production, and cell survival. This complex is ubiquitous across nearly all animal cell types and plays a critical role in a multitude of cellular processes [99]. Typically, NF-κB proteins form homodimers or heterodimers composed of p65 and p50 subunits. In the cytoplasm, these dimers bind to the inhibitory protein IκB, forming an inactive trimeric complex. The activation process begins when upstream signaling molecules, such as TNF, bind to their respective receptors on the cell membrane. This binding induces a conformational change in the receptor, which then transmits a signal to IκB kinase (IKK). IKK, in turn, phosphorylates IκB, leading to its dissociation from the trimeric complex [100]. The free NF-κB dimer then translocates to the nucleus, where it binds to specific DNA sequences, initiating the transcription of genes that are pivotal for cell proliferation and survival, such as Cyclin D1, c-MYC, MMP-9, and VEGF. The sustained activation of NF-κB can thus result in uncontrolled cellular growth. Moreover, the activation of NF-κB can stimulate antiapoptotic proteins such as IAPs, promote EMT in GSCs, and induce chemotherapy resistance in GBM [101]. 

NF-κB also plays a key role in modulating the immune response to infections. Aerobic glycolysis facilitates the upregulation of PD-L1 expression by NF-κB, contributing to the immune evasion observed in GBM [ 102, 103] . Additionally, NF-κB is involved in the regulation of METTL3, enhancing GBM proliferation, migration/invasion, and tumor malignancy [104]. Interactions of NF-κB with noncoding RNAs have been shown to amplify the Warburg effect and angiogenesis, correlating with a poorer prognosis in GBM patients [105]. In conclusion, NF-κB is a central regulatory nexus in the progression of GBM. 

### HIF-1α

To adapt to hypoxia, GBM cells can express a TF known as hypoxia-inducible factor (HIF), which can promote the dedifferentiation of differentiated glioma cells and induce the formation of GSCs. However, only CSCs lead to the progression of malignant gliomas, ultimately resulting in a poor prognosis [4]. HIF is a heterodimer composed of α-subunits (HIF-1α, HIF-2α, or HIF-3α) and a β-subunit (HIF1-β). HIF-1α is ubiquitous and a key molecule in the hypoxia regulation of CSCs [106]. HIF-1α appears to bind to the promoter of *CD133* (a marker gene of CSCs), promoting the production of CD133 ^+^ glioma stem-like cells through OCT4 and SOX2. In turn, CD133 promotes the expression of HIF-1α and its translocation to the nucleus under hypoxic conditions [107]. It has also been found that the proportion of undifferentiated CD133 ^+^ glioma cells increases under hypoxic conditions. Blazek *et al*. [107] and Platet *et al*. [108] focused on analyzing this phenomenon because hypoxia affects the frequency of symmetric and asymmetric divisions of GSC subcellular stemness characteristics, leading to an increased ratio of newly formed GSCs in the tumor. In addition, differentiated glioma cells extracted from GBM-derived neurospheres can be induced to differentiate into CD133 ^+^ GSCs under hypoxic conditions [4]. Generally, CD133 plays a role in promoting cancer in GBM, but the function of CD133 seems to vary at different levels of glioma. A study by Wu *et al*. [109] indicated that high CD133 expression is associated with worse overall survival in patients with WHO grade IV neuroglioma but is not associated with outcomes in patients with WHO grade II–III tumors. This is an intriguing phenomenon. However, to date, no studies have elucidated the differences in CD133 function across different grades of glioma. 

HIF-1α in GBM possesses unique and sometimes paradoxical features. On the one hand, in line with the findings of Platet *et al*. [108], HIF-1α/HIF-2α under hypoxic conditions induces the dedifferentiation of glioma cells into CSCs through Sox2 [4]. Under hypoxia, increased HIF-1α in GBM cells activates the HIF-1α-SERPINE1 pathway, JAK1/2-STAT3 pathway, and Notch signaling pathway, contributing to therapeutic resistance and malignancy [ 110, 111] . Concurrently, targeted silencing of *HIF-1α* using siRNA can enhance the radiosensitivity of malignant gliomas [112]. There is substantial evidence supporting the role of HIF-1α in promoting the progression and metabolism of GBM [113]. Conversely, the balance of HIF-1α/Wnt gene transcription signaling triggered under hypoxic conditions is responsible for the transcriptional regulation of the phenotypic transition of GBM stem cells toward neuronal differentiation [114]. HIF-1α is significantly associated with IDH1/2 mutations, which predict a better prognosis for GBM patients [115]. Roxadustat, a small-molecule stabilizer of HIF-1α that inhibits prolyl hydroxylase (PHD), amplifies HIF-1α signals, significantly inhibits the growth of GBM cells, and extends the survival of mice with chemoresistant GBM without apparent organ toxicity [116]. The role of HIF-1α in the development of GBM and within the hypoxic tumor microenvironment is highly complex. For example, HIF-1α regulates angiogenesis, metabolic reprogramming, and transcriptional signaling pathways such as the EGFR, PI3K/Akt, and MAPK/ERK pathways. It influences cell migration and invasion by regulating glucose metabolism and growth in GBM cells [ 117, 118] . HIF-1α also regulates ABCG2 and MGMT, affecting sensitivity to the chemotherapeutic drug TMZ [119]. In summary, the function of HIF-1α in specific contexts depends on the balance between its tumor-suppressive and oncogenic properties. 

### KLF4

KLF4 primarily functions to regulate cellular proliferation and differentiation, maintains the normal state of cells and even has the ability to suppress tumor development. However, GBM cells are aberrantly expressed [ 120, 121] . Ma *et al*. [121] discovered that KLF4 binds to the promoter of *ITGB4*, upregulating its expression, which sustains the self-renewal and stemness of GSCs and is significantly correlated with increased tumor grade. Through the suppression of KLF4 expression by miR-152 or the enhancement of KLF4 expression by miR-92a, KLF4 is a critical factor in the proliferation and invasion of GBM tumor cells [ 122, 123] . KLF4 can also increase the spare respiratory capacity of GBM cells by inducing mitochondrial fusion, providing additional nutrients for proliferation and survival [124]. Under hypoxic conditions, KLF4 supports the malignant progression of GBM through the EGFR-PI3K/AKT signaling pathway [125]. However, the mechanisms of KLF4 in GSCs, including its relationship with the survival of GBM patients, are not entirely clear. Nonetheless, there is no doubt that KLF4 is a potential target for therapeutic intervention in GBM. 

### STAT3

STAT3 is a critical player in cancer biology, particularly in GBM, where it influences invasion, cell cycle regulation, and immune system resistance. It is pivotal for the self-renewal and differentiation of tumor stem cells and participates in various signaling pathways that regulate GSCs. For instance, Chi3l1, a protein associated with GBM, interacts with CD44 on GSCs, activating STAT3, among other pathways, which drives GSCs toward a mesenchymal expression profile and enhances self-renewal [126]. Similarly, adenovirus infection of glioma cells promotes GSC formation via the TLR9/NEAT1/STAT3 pathway [127]. STAT3 also contributes to tumor cell invasion and cell cycle regulation through mechanisms such as TGFBI secretion by tumor-associated macrophages (TAMs) and ARPC1B activation, which supports mesenchymal phenotype maintenance and radiotherapy resistance in GSCs [ 128– 130] . In the context of immune resistance, STAT3 establishes an immunosuppressive tumor microenvironment, with processes such as CXCL8 maintaining the mesenchymal state of GSCs and inducing M2-like TAM polarization and the TFPI2-CD51-STAT6 axis facilitating immunosuppressive microglial polarization [ 131, 132] . The complexity of the functions of STAT3 in GBM underscores its potential as a therapeutic target, with strategies aimed at inhibiting its pathways showing promise in curtailing the formidable self-renewal and tumorigenicity of GSCs, thereby offering a new avenue for overcoming therapeutic resistance in this aggressive cancer. 

### β-Catenin

In the intricate landscape of GBM, β-catenin plays a critical role in the Wnt signaling pathway, driving the stem-like properties that fuel tumor growth and resistance. The dysregulation of this pathway not only accelerates tumor proliferation but also enhances the adaptability of cancer cells, making conventional treatments challenging. A prime example of the multifunctionality of β-catenin is the role of WISP1, a protein secreted by GSCs that sustains both GSCs and tumor-supportive macrophages through its interaction with integrin α6β1-Akt, contributing to the integrity of the tumor microenvironment [ 133, 134] . The targeted inhibition of this signaling axis, notably by compounds such as carnosic acid, has emerged as a promising therapeutic approach. Furthermore, the protein Chi3l1, which is prevalent in GBM, influences the GSC state by engaging with CD44, triggering a cascade involving β-catenin, Akt, and STAT3, propelling GSCs toward a mesenchymal phenotype and indicating the critical influence of β-catenin on GSC plasticity and tumor progression [135]. The development of small-molecule inhibitors targeting the Wnt/β-catenin pathway represents a notable advancement in cancer therapeutics, showing efficacy in interrupting the cancer cell cycle, curbing proliferation, and bolstering immune responses [ 136– 140] . In the context of radioresistance, the role of β-catenin is underscored by the increased expression of N-cadherin in GSCs, which leads to the accumulation of β-catenin and the suppression of proliferative signaling [ 133, 134, 141, 142] . Under hypoxic conditions, the activity of the glycosyltransferase GLT8D1, which promotes Wnt/β-catenin signaling, illustrates the complexity of tumor microenvironment interactions, which are linked to more aggressive glioma grades and poorer clinical outcomes [ 143 – 145] . In summary, the various roles of β-catenin, spanning from maintaining tumor stem cell populations to influencing the immune milieu and dictating treatment responses, make it a crucial target for innovative therapeutic strategies, aligning with the current paradigm shift in oncology emphasizing the need for targeted molecular therapies to effectively manage complex malignancies such as GBM. 

### YAP/TAZ

In the evolving field of GBM research, YAP/TAZ, which are integral to the Hippo pathway, have attracted increasing attention for their complex roles in gene regulation, cell growth, apoptosis, and stem cell renewal [146]. These coactivators are pivotal in balancing GSC self-renewal and differentiation, thereby maintaining stemness and promoting tumor growth. Moreover, YAP/TAZ are known to enhance GBM malignancy by promoting tumor invasion and rapid cell cycling in GSCs and by modulating interactions with crucial pathways such as the EGFR and Wnt pathways [ 147, 148] . This interaction extends to modulating the tumor immune response, with emerging evidence highlighting the role of YAP/TAZ in immune evasion. Preclinical models have shown promising results when targeting YAP/TAZ with drugs, such as verteporfin, which disrupt the YAP-TEAD interaction, and their potential to inhibit GBM growth and enhance susceptibility to immune-mediated destruction has been explored [ 149– 152] . This indicates that YAP/TAZ are key obstacles in GSC differentiation and immune evasion therapy, and future research focusing on exploring the complex interactions of YAP/TAZ with multiple cellular pathways and the immune system, as well as developing more targeted and effective therapeutic strategies, will be of vital importance. 

## Conclusions and Prospects

In conclusion, the role of TFs in the regulation of GSCs represents a pivotal axis in the pathobiology of GBM. The intricate network of TFs, including SOX2, OCT4, NANOG, KLF4, c-MYC, β-catenin, STAT3, NF-κB, HIF-1α and YAP/TAZ, is central to the maintenance of the stem-like properties of GSCs, which in turn drive tumor heterogeneity, therapeutic resistance, and recurrence. The expression levels of these TFs correlate with GBM progression and patient prognosis, highlighting their potential as biomarkers and therapeutic targets. Since the levels of TFs in normal tissues are within controllable ranges but are highly expressed in GBM, directly targeting TFs therapeutically may lead to severe, unnecessary side effects. Strategies such as encapsulating targeted inhibitors in physical materials can highlight the advantages of TF-specific cancer treatment. Moreover, the specificity of TFs for GSCs versus normal stem cells further complicates the therapeutic landscape, necessitating the development of highly selective TF modulators.

Future research directions must focus on the comprehensive elucidation of the TF regulatory networks within GSCs. This will require advanced genomic and proteomic approaches to map the interactions and effects of TFs on GSC behavior and their microenvironment. The development of novel therapeutic strategies targeting these TFs will depend on our ability to selectively disrupt their regulatory functions within GSCs. Such strategies may include small molecule inhibitors, monoclonal antibodies, or gene therapy approaches designed to modulate TF activity or expression. The ultimate goal is to translate these insights into clinically effective treatments that can improve survival outcomes for patients with GBM, a goal that remains one of the most challenging issues in oncology.
